# Presentation and Management of Klippel-Trenaunay Syndrome: A Review of Available Data

**DOI:** 10.7759/cureus.8023

**Published:** 2020-05-08

**Authors:** Fahham Asghar, Ramsha Aqeel, Umar Farooque, Aatera Haq, Muhammad Taimur

**Affiliations:** 1 Neurology, Dow Medical College, Dow University of Health Sciences, Karachi, PAK; 2 Internal Medicine, Dow Medical College, Dow University of Health Sciences, Karachi, PAK; 3 Surgery, Civil Hospital, Karachi, PAK; 4 Internal Medicine, Dow University of Health Sciences, Karachi, PAK

**Keywords:** klippel-trenaunay syndrome, varicose veins, hemangioma, laser treatment, rare disease, bone hypertrophy, port-wine stain

## Abstract

Klippel-Trenaunay Syndrome (KTS) is a rare and sporadic congenital disorder, characterized by the classical triad of port-wine stains, varicosities along with bone and soft tissue hypertrophy. Symptoms of Klippel-Trenaunay Syndrome include pain, swelling, lymphedema, bleeding, superficial thrombophlebitis, and deep vein thrombosis. The etiology remains indistinct and has been attributed to both genetic and environmental factors. In most cases, a thorough history and clinical examination is enough for the diagnosis of Klippel Trenaunay Syndrome. However, when certain complications are present, noninvasive imaging techniques are used for the diagnosis and evaluation of the disease in patients. Due to the diversity of presentation, a multidisciplinary approach is essential for the proper management of such patients. At present, there is no cure for the disease; rather, symptomatic treatment is employed in order to improve the patients' quality of life. In this review, we provide a brief overview of the clinicopathological profile and management of Klippel-Trenaunay Syndrome.

## Introduction and background

Klippel-Trenaunay Syndrome was first described in the year 1900 by two French physicians, Maurice Klippel and Paul Trenaunay, in two patients with hemangiomatous lesions of the skin associated with asymmetric soft tissue and bone hypertrophy [1]. It is also referred to as "angioosteohypertrophy syndrome" and "hemangiectatic hypertrophy." Klippel-Trenaunay Syndrome has been listed as a “rare disease,” which presents at birth, early infancy, or childhood. The rarity of the disease is a major hurdle in the calculation of the incidence of KTS. Nevertheless, the incidence has been estimated at two to five per 100,000 [2-3]. KTS is reported more often in males than in females [3]. However, no racial predilection has been observed for the syndrome.

## Review

Diagnosis

Being an extremely rare disease, there are no formally described definite diagnostic criteria nor any pathognomonic laboratory test investigation for this syndrome. The diagnosis is generally dependent on two of the following features of the classical triad of KTS [4]:

Cutaneous hemangiomas (port-wine-stains) Varicosities Bone and soft tissue hypertrophy The distribution of these symptoms in patients has been described by various studies. A study conducted with 768 patients reported the elongation of the impaired limb in all patients, edema in 84%, varicose veins in 36%, and flat angiomata in 32% [5]. Another study conducted in 1991 on KTS reviewed 144 patients, of which 95.1% had a cutaneous vascular malformation, 93.1% had soft tissue or bony hypertrophy, and one lower extremity was affected in 71.5% [6]. Another study found capillary malformations in 98%, varicosities in 72%, and limb hypertrophy in 67%, with all three features being present in 63% of the patients [7]. Malformations like port-wine stains, lymphedema, lymphangiomas, and arteriovenous fistulas are associated with KTS, but it is described as a complex, slow-flow vascular malformation [8-9].

Clinical manifestations

According to the Hamburg classification of vascular malformation, KTS has been classified as a mixed type of vascular abnormality that is composed of capillary, lymphatic, and venous malformations [10]. It is unilateral and mostly affects the lower extremities; rarely, it can affect bilateral limbs and the upper extremities. The common symptoms are pain and lymphedema [11]. There are other, various symptoms of KTS, including superficial thrombophlebitis, deep vein thrombosis, pulmonary embolism, bleeding, hypospadias, oligodactyly, paresthesia, angiosarcoma, ulceration, thrombosis, stasis dermatitis, decalcification of involved bones, poor wound healing, hyperhidrosis, hypertrichosis, and spina bifida.

*Cutaneous Hemangiomas (Port-Wine Stain)* Vascular malformations can be divided into two main categories: vascular dysmorphias and tumors (hemangiomas) [12-13]. Vascular dysmorphias are developmental abnormalities that can be classified as lymphatic, arterial, venous, and arteriovenous malformations [12]. The port-wine stains/capillary hemangiomas are mostly present at birth, with a patchy distribution appearing first. They are present as red or purple marks that are irregular but may have a comparatively linear border. Sharp demarcation is noticed if they are present on the trunk. It can be distributed in a confluent geographic pattern or more randomly on the affected limb and adjacent trunk. The presence of a geographic vascular stain is a predictor of the risk of associated lymphatic malformation and certain complications like sepsis, cellulitis, and phlebitis in patients with KTS [14]. Large cutaneous hemangiomas sequester platelets ending up in a consumptive coagulopathy are known as Kasabach-Merritt Syndrome [4,15]. Vascular malformations can also involve visceral organs such as the spleen, liver, pleura, bladder, and colon. Visceral organ involvement causes internal hemorrhage and may manifest as hematuria or hematochezia.

*Varicosities* Varicose veins are present since birth. They affect extensive areas of the extremities. They may remain static or progress at a slow pace. These varicosities can be found below the knee, above the knee, and sometimes in the pelvic region. The deep and perforating veins can also be involved. The deep venous system of the affected extremity can undergo hypoplasia, agenesis, atresia, duplication, and abnormal venous valve formation, ending up in an abnormal venous system [7,16-18]. Various structures like fibrous bands, abnormal muscles, aberrant arteries, and venous sheaths are known to compress the deep veins. The varicosities result in a “Klippel-Trenaunay vein,” which can be present at birth or not become prominent until the child begins to walk. This vein starts in the foot or lower leg and increases proximally, entering the thigh or the gluteal area [4,15].

*Bone & Soft tissue Hypertrophy* The third aspect of the triad, skeletal, and soft tissue hypertrophy is an important characteristic of the syndrome. Mostly, a single lower extremity is involved [12,16-17]. The increased length of the limb is associated with bone hypertrophy while the increase in girth is due to soft tissue hypertrophy. The possible mechanisms behind the overgrowth of the affected limb have been poorly defined and include girth increase by venous hypertension, limb elongation, and atresia of the venous system, leading to stasis, edema, and varicosities, increased blood flow through the abnormal capillary network, cutaneous venous channels causing overgrowth in fetal life, defective remodeling of vessels during embryogenesis, and mesodermal defect-inducing hemangiomas and varicose veins [14,16]. Limb discrepancies of as much as 12 cm have been noted [4,11]. Craniofacial involvement can also occur in this syndrome, leading to a deviated nasal septum, nasal obstruction, oral and nasal mucosa having angiomatous malformations, leading to intermittent nasal bleeding. There can also be jaw enlargement, facial asymmetry, premature tooth eruption, and hemangiomas of lips and tongue [4]. Furthermore, brain abnormalities, including hemorrhage, hemimegalencephaly, hydrocephalus, infarction, and seizures, can occur [19-21]. Syndactyly, macrodactyly, polydactyly, and hip dysplasia have also been reported in about 29% of patients with KTS [6]. *Complications* Several complications have been reported in KTS. Intestinal lymphangiectasia causes protein-losing enteropathy. Pain, bleeding, and diarrhea occur due to gastrointestinal varicose [4,22]. The involvement of the gastrointestinal tract (GIT) may be unnoticed in patients without symptoms. GI bleeding usually starts in the first decade of life and the most commonly reported source is the diffuse cavernous hemangiomas of the distal colon and rectum. These occur in an estimated 1%-12.5% of KTS cases [23,24]. Upper gastrointestinal bleeding also occurs due to hypoplasia of the portal vein causing portal hypertension leading to esophageal varices [23,25]. Although uncommon, genitourinary tract anomalies may occur as vascular malformations in the bladder, scrotum, penis, vulva, and vagina [26]. Abnormal lymphatic drainage can cause infection cellulitis and ulceration of the affected limb. Skin tumors of the affected limb are also noticed, including basal cell carcinoma and squamous cell carcinoma, which are due to long-standing venous ulcers. Venous thromboembolism is also a complication that can end up in pulmonary hypertension and subsequent right heart failure [4]. Complications occur in pregnancy, which depends on the location of the vascular abnormality. A hypercoagulable condition in pregnancy leads to increased morbidity. The risk for intrauterine growth retardation also rises [4,27]. Venous insufficiency and lymphedema cause pain, which is the most common problem in this syndrome [16]. Thrombophlebitis and gangrene are also two of the few local complications in KTS. The involvement of the internal organs causes neurovascular anomalies, pulmonary vein varicosities, pleuro-pericardial effusions, pulmonary embolism, and colorectal and urinary tract hemorrhage [22].

Proposed pathogenesis

The etiology of KTS remains indistinct. Its incidence is sporadic; however, unproven autosomal dominant inheritance has also been reported [12]. We now describe the proposed pathogenesis of the disease.

Deep Vein Atresia

Studies have suggested that atresia or obstruction of any of the deep veins in the leg produces chronic venous hypertension, which in turn produces nevus, varices, and hypertrophy, which are the cardinal signs of KTS [28-29].

Chronic Venous Hypertension

In 1945, Servelle studied venograms of KTS patients and concluded that if the main vein of a limb is ligated, the resulting venous stasis produces an elongation of that particular limb [5]. He also proposed that the abnormalities in KTS are due to chronic venous hypertension secondary to deep vein abnormality or obstruction [30]. Servelle was opposed by a study in which the results showed normal deep veins in 60% and normal calf pump function in 84% of patients with KTS [13].

Persistence of Embryological Vascular System

Bourde suggested the persistence of part of the embryological vascular system as the underlying cause [31]. However, many of the associated conditions of KTS such as hypospadias and Syndactyly cannot be caused by a solely vascular abnormality. *Mesodermal Anomaly*

Later, Young suggested that the association of KTS with bone and soft tissue abnormalities points towards a generalized developmental mesodermal anomaly [32]. A mesodermal defect, which acts primarily on angiogenesis, could explain all the features of this condition. If the embryonic vascular reticular network in the developing limb bud regressed later than usual, the developing limb would be subjected to an increase in skin blood flow leading to hypertrophy and hyperplasia of the veins. This, in turn, increases the rate of bone growth causing histological changes of intimal thickening, elastosis, and ectasia that are found in the superficial veins after birth, and are clinically apparent as a skin hemangiomas and varicose veins [29-34].

Management 

Since the facts regarding the etiology of KTS have not yet been established fully, no consensus has been achieved for its proper treatment or approved management. Data addressing the issue of management are scarce and focus on only a few important points. Due to the diversity of involved structures, various problems faced by the patients, ranging from an abnormal walking posture to psychological disturbances, and the vast differences in age groups with the disease, a multidisciplinary approach is essential for the proper management of these patients [2,14,35-37]. At present, there is no cure for the disease; rather, symptomatic treatment is employed in order to improve the patients' quality of life. Relative indications for intervention include pain, functional impairment, swelling, limb asymmetry, or cosmetic reasons. Absolute indications include hemorrhage, infections, refractory ulcers, or acute thromboembolism [36]. *Conservative Managemen*t

A conservative approach should be employed towards the symptomatic treatment of KTS by the use of non-invasive measures. In the majority of the cases of KTS, the patient is advised to wear elastic or non-elastic compression stockings. Elastic stockings combined with psychological support have been found to be the most effective approach in the management of patients with KTS [6,18]. These stockings are coupled with other conservative measures such as frequent leg elevation, physiotherapy, lifestyle modifications, and maintenance of strict hygiene. The use of analgesics, antibiotics, and corticosteroids is commonly employed for treating cellulitis and thrombophlebitis. Anticoagulants can be used, either prophylactically, before surgery, or in cases of acute thrombosis [14,38]. Conventional sclerotherapy, although a good option for small malformations, is ineffective in the management of larger malformations [38].

*Pain Management* Pain is a common feature of KTS, affecting as many as 88% of those affected [2]. Therefore, patients with KTS require pain management frequently. The guiding principles of management are dependent on the cause of pain and, therefore, causative treatment is employed in the treatment of pain in these patients [2]. Pain arising as a result of chronic venous insufficiency is managed via compression stockings and/or surgically. Pain due to recurrent cellulitis is managed by the judicious use of antibiotics and analgesics and via maintenance of strict hygiene [2] Pain as a result of intraosseous vascular malformations requires surgical intervention. In cases where the lesions cannot be removed, long-term treatment with appropriate analgesics, such as opiates may be employed [2]. Neuropathic pain is, in the majority of cases, refractory to conventional analgesics. In such cases, antidepressants and anticonvulsants may be employed. A multi-drug regimen may be needed, and a short-term course of steroids may be deemed necessary [2].

Surgical Management

Surgery is usually reserved for symptomatic cases. The success of surgery depends on the pre-operative evaluation of the deep venous system using techniques such as computed tomography (CT) arteriography, duplex scanning contrast geography, which can help ascertain the extent of vascular involvement, and the presence of arteriovenous fistulae. Many authors have also reported worsening of the symptoms following procedures such as multiple ligations and stripping [39].

Laser Treatment and Radiotherapy

The treatment of port wine stain often involves the use of laser treatment [36-37]. Laser therapy can also be employed in cases of ulceration. Raised lesions or lesions under the skin usually do not respond well to laser treatment [37]. Endovenous laser therapy on the greater saphenous veins can also be used in patients with KTS for the management of varicosities. Radiotherapy has been reported to be of help in some cases of KTS; the radiation may help to induce regression of hemangiomas. However, the results are usually slow to develop [40].

## Conclusions

Being a rare disease, research work on Klippel-Trenaunay Syndrome is scarce. Most of the researches published report conflicting results regarding its modes of inheritance, pathogenesis, and effective management. The autosomal dominant mode of inheritance is suggested by a few researchers; others have shown KTS to be caused by a single gene defect, while a few have not shown any definitive gene localization. Out of the various pathogenic causes, a mesodermal defect affecting angiogenesis as the main cause underlying the disease can explain the majority of the symptoms of the disease, but it has not been well-established. Klippel-Trenaunay Syndrome is mostly described by researches with a limited number of patients, from different parts of the world, with multiple suggested ways of management, rather than following a well-defined, established mode of treatment. It is imperative that more studies be done to improve the quality of life of patients with this rare disorder.
